# A study of the effects of olfactory stimulus duration and concentration on sleep arousal under fatigue driving

**DOI:** 10.3389/fpsyg.2025.1704018

**Published:** 2025-11-27

**Authors:** Faren Huo, Yingfan Lu, Fei Fang, Xuanhui Liu

**Affiliations:** Pan Tianshou College of Architecture, Art and Design, Ningbo University, Ningbo, Zhejiang, China

**Keywords:** sense of smell, fatigue coping strategies, driving fatigue, sleepiness, driving simulator

## Abstract

Olfactory stimulation can alleviate passive task-related fatigue (PTR), but its mechanism and influencing factors are still unclear. This study used a controlled experimental design to explore the effect of mint odor on driving fatigue by comparing the driving fatigue induction stage (monotonous driving) with the awakening stage (olfactory intervention). The experiment controlled the stimulation duration (1 min vs. 8 min), traffic flow (low vs. high), and concentration (low, medium, and high), and combined subjective scales, heart rate variability (HRV), and percent eyelid closure (PERCLOS) indicators to evaluate the effect. The results showed that under high traffic flow, 8 min of stimulation relieved fatigue most significantly; moderate concentration was the best for physiological arousal, but high concentration was subjectively preferred; HRV was more sensitive than PERCLOS, and age and driving experience regulated the response. Context-based olfactory intervention can effectively alleviate fatigue, and stimulation duration and environmental load are key regulatory factors. This study advances the theoretical framework of multimodal fatigue management and provides practical suggestions for the design of adaptive vehicle warning systems.

## 1 Introduction

Driving fatigue is a major factor in fatal car accidents, and relevant experts believe that 20–30% of traffic accidents are caused by driving fatigue (Hashemi Nazari et al., 2017). Most drivers still choose to continue driving when they feel sleepy (Zwahlen et al., 2016), which can lead to traffic accidents, causing a series of hazards or even loss of life. Therefore, the consequences of driving fatigue need to be addressed at the source. How can we alleviate or prevent driver fatigue? Finding the most effective solutions requires multifaceted research.

Current research explores the effectiveness of combating driver fatigue from different dimensions. In the visual dimension, one study revealed the limitations of single visual cues in regulating driving behavior (McIlroy et al., 2017). Pure visual information is less effective in promoting ecological driving behavior, especially in inhibiting rapid acceleration behavior, and its intervention is significantly weaker than auditory and tactile modalities, a difference that may stem from the fact that visual information is more likely to be ignored. In the auditory dimension, music was found to have a transient positive effect on driving fatigue, but the effect was limited, and driving fatigue still occurred after 15–25 min of driving. This study was only conducted with young people (Orsini et al., 2024); when driving for longer periods, drivers entered into a state of fatigue irrespective of whether the driving context included different mood music (positive or negative) (Hu et al., 2022). In the haptic dimension, additional torque stimulation applied through the steering wheel was effective in improving the handling performance of fatigued drivers and significantly reduced the level of passive fatigue. However, this study was only conducted on males, and the effectiveness of this intervention has not been verified for female drivers (Wang et al., 2017). In addition, it was found that the tactile guidance provided by the accelerator pedal, while effective, was no different from auditory stimulation (McIlroy et al., 2017). In the audiovisual dimension, visual and auditory stimuli are less satisfying, acceptable, and effective for users than haptic stimuli (McIlroy et al., 2017). Some studies have found that multimodal warnings are superior to unimodal warnings in driving (i.e., modal effects) and that there is a mapping effect in audiovisual warnings, where only a high mapping auditory component facilitates warning effectiveness; however, a high mapping component may distract the driver from the road (Wang et al., 2022). Although existing studies have extensively explored the mitigation of driving fatigue in visual, auditory, and haptic dimensions, several limitations remain. First, visual information is easily overlooked, the effect of auditory stimulation is short-lived, and gender differences in tactile stimulation have not been fully investigated. Second, multimodal stimuli, although superior to unimodal stimuli in some cases, are still controversial in terms of their acceptability and efficacy and may distract drivers’ attention. In addition, existing studies have focused on visual, auditory, and tactile dimensions, while olfaction, as a potential and under-explored sensory dimension, has not been systematically investigated for its effects on driving fatigue. The unique advantages of olfaction, such as not being easily overlooked, not requiring active attention, and being able to sustain its effects, make it potentially applicable in alleviating driving fatigue. Therefore, this study aims to fill this research gap, explore the effects of olfactory stimuli on driving fatigue and its mechanisms, and provide new perspectives and methods for multimodal driving fatigue intervention.

Odors affect the human body through two main pathways: olfactory and trigeminal (Shusterman and Hummel, 2009). Aromatherapy has been shown to alleviate depressive emotions in drivers and positively impact stress reduction at work (Seo et al., 2020). In the field of driving, specific scents have demonstrated significant physiological modulating effects: peppermint effectively suppresses drowsiness (Norrish and Dwyer, 2005); rosemary increases driver alertness (Yoshida et al., 2011); and lemon helps maintain driving concentration (Martial et al., 2023). In addition, citrus aroma can reduce exercise fatigue (Kwon et al., 2020); lavender aroma can reduce occupational fatigue; and woody aroma has the effect of moderating negative emotions (Yu et al., 2022). At the physiological level, olfaction can regulate emotions, affect neurological and cognitive functions, and act on a variety of physiological indicators such as heart rate, blood pressure, and brain waves (Yu et al., 2022), resulting in physiological responses. Humans can also use smell to influence alertness and fatigue to some extent (Herz, 2009; Kontaris et al., 2020). On the psychological level, specific odor environments can evoke relevant memories and experiences, stimulate positive emotions and attention, thereby increasing alertness and reducing fatigue (Epple and Herz, 1999). In terms of olfactory duration, it has been shown that both short- and long-term exposure to peppermint odor can increase driver awareness, with no significant difference in effects between the two exposure conditions (Pujiartati et al., 2019). However, one study found that a 1-min release of the odor followed by a fluctuating release between 5 and 9 min was the most effective method (Yoshida et al., 2011). Therefore, the effect of olfactory duration still needs further exploration. Regarding odor concentration, the results of the study showed (Baccarani et al., 2021) that for all odors, higher concentrations enhanced perceptual intensity and stimulation judgments, but were not associated with lower relaxation judgments. In terms of external stress, the study found that consumers in the low arousal odor condition perceived shorter durations than those in the high arousal odor condition, but concluded that the effect of odor arousal on time perception was attenuated in high-stress conditions (Yu et al., 2024).

However, the specific role of olfaction in the hedonic experience of driving needs further investigation, and the effects and roles of olfactory stimuli on fatigue arousal under the influences of concentration, duration, and external pressure are not yet clear. Therefore, we investigated the effects of olfactory stimulus duration and concentration on sleep arousal during fatigue driving. A comprehensive understanding of the different sensations and effects of various olfactory stimuli on sleep arousal in fatigue situations can provide further guidance for effective countermeasures against driving fatigue.

## 2 Related works

### 2.1 Driving fatigue

Studies have shown that driver fatigue is a significant factor in 10–30% of fatal crashes (Sjörs Dahlman et al., 2024). Fatigue is divided into two main categories: sleep-related fatigue and task-related fatigue. The former is influenced by sleep deprivation, wakefulness duration, and circadian rhythms, and its subjective sleepiness is significantly correlated with lane departure behavior (Åkerstedt et al., 2014). The latter includes active task-related (ATR) fatigue and passive task-related (PTR) fatigue (May and Baldwin, 2009; Phillips, 2015). Notably, PTR fatigue alone reduces alertness, manifesting itself in slower braking and steering responses to emergencies, increasing crash risk (Saxby et al., 2013).

Research has shown that when odor delivery parameters are precisely controlled, olfactory cues can improve driving behavior by providing a comfortable interactive experience without distraction (Herz, 2004). This intervention can effectively complement visual cues, and its mechanism of action is rooted in the emotion-regulating function of odors. Specifically, different odors elicit different pleasurable feelings (Baeyens et al., 1996), and this hedonic response, which is learned through emotional associations (Herz, 2004), may be potentially effective in reducing driving fatigue. As a complementary treatment, aromatherapy relieves stress, anxiety, and even induces feelings of pleasure by rapidly modulating emotional states. Essential oil components promote relaxation (e.g., ylang-ylang essential oil has been shown to have hypotensive and calming effects) while maintaining necessary levels of alertness. These findings provide an important basis for exploring the optimal use of olfactory interventions in driving fatigue management (Jung et al., 2013).

### 2.2 Fatigue awakening

Optimal arousal theory (OAT) (Dodson, 1915) states that cognitive task performance peaks when an individual is in an optimal state of arousal, whereas too low or too high a level of arousal results in decreased performance due to inertia and anxiety, respectively. Herb’s further development of OAT emphasizes that individuals’ “cueing function” is optimal during optimal arousal and that this relationship is not affected by irrelevant factors such as task difficulty. Significant differences in optimal arousal point and arousal stimulus tolerance were found between individuals.

Berlyne and Boudewijns (1971), based on Wundt’s bell curve theory, proposed that arousal level has an inverted U-shaped relationship with hedonic experience, with pleasure and preference maximized when the arousal level is at an optimal state. This hedonic experience at optimal arousal can be considered a reference point for optimal arousal (Berlyne and Boudewijns, 1971; Chmiel and Schubert, 2017). Studies have shown that odors have significant emotional arousal properties (Vernet-Maury et al., 1999), where pleasant odors induce positive emotions and unpleasant odors trigger negative emotions (Epple and Herz, 1999).

According to existing research, olfactory stimulation may produce an optimal pleasure experience by modulating psychophysiological arousal levels. The key issue is to explore whether different concentrations and durations of peppermint aroma can effectively enhance the level of sleep arousal, which provides an important basis for investigating the effects of olfactory stimulation on sleep arousal.

Therefore, it is necessary to investigate the significant effects of olfactory stimuli on sleep arousal.

### 2.3 Odor concentration and duration of arousal

Regarding the effects of odor concentration, studies have shown a significant dose–effect relationship. Baccarani et al. found that odor concentration was positively correlated with perceived intensity and level of stimulation, but not with the relaxation field (Baccarani et al., 2021). One study (Li et al., 2023) showed that both pleasant and unpleasant odors showed intensity desensitization, with pleasant odors being more susceptible to emotional habituation and unpleasant odors being more desensitized at low concentrations. Jin et al. showed that the emotional attributes of odors shifted with concentration, from “calm-relaxed” at low concentrations to “euphoric-energetic” at medium to high concentrations, and that this shift occurred at medium to high concentrations. This shift may be related to the stimulant properties of odors at high concentrations (Jin et al., 2018). Li et al. also found that the “arousal dimension” of odors is directly related to their perceived intensity and is odor-specific (Li et al., 2023). However, that different concentrations of the same odor were similarly rated for pleasure and familiarity within the tolerable concentration range and were less likely to have a significant effect on physiological indicators. These studies suggest a complex non-linear relationship between odor concentration and fatigue relief effects. Therefore, it is necessary to investigate the significant effects of the concentration of olfactory stimuli on arousal from fatigue driving.

Regarding the duration of olfaction, studies have shown that intermittent release patterns (fluctuating 5–9 min after the initial 1 min) are more effective (Yoshida et al., 2011), while continuous olfactory stimulation leads to a decrease in perceived intensity (Hummel and Kobal, 1999; Pellegrino et al., 2017). Over time, drivers may become acclimatized to the odor and diminishing the effect (Jellinek, 1997; Hongratanaworakit et al., 2004). However, there is no significant difference between the effects of short- and long-term exposure to short mint odor (Pujiartati et al., 2019). Another study showed that the longer the odor stimulus appeared, the greater the concentration perceived by participants (Tang et al., 2019). Therefore, it is necessary to investigate the effect of the duration of olfactory stimuli on arousal to fatigue driving.

In terms of environmental stress, it is mainly presented as traffic flow size, which is an important environmental factor affecting driving fatigue, while the effect of odor stimulation may vary depending on the traffic flow. Both monotonous road environments and high traffic flow environments have been found to contribute to driving fatigue, but fatigue is more pronounced in high traffic flow environments because drivers need to continuously cope with frequent traffic changes and decision-making demands (Thiffault and Bergeron, 2003). Matthews and Desmond (2002) suggest that driving tasks in high traffic flow conditions lead to higher mental loads, which accelerate fatigue. Conversely, in low traffic flow or overly monotonous road conditions, drivers may experience burnout due to a lack of stimulation, leading to a decrease in attention and an increased risk of fatigue. Therefore, it is necessary to investigate the effects of olfactory stimuli on arousal related to fatigued driving when traffic flow varies.

### 2.4 Research question and hypotheses

First, fatigued driving, as an important hidden danger of road traffic safety, has limited effects from existing interventions; second, although olfactory stimulation has been proven to alleviate passive task-related (PTR) fatigue, its mechanism of action and influencing factors are still unclear; lastly, the intervention effect of olfactory stimulation parameters (concentration, fatigue awakening time, etc.) on driving fatigue still needs to be systematically verified. This study presents key questions based on the above research background:

- Q1: Is the effect of olfactory stimulation on the reduction of driving fatigue statistically significant?
- Q2: Are there significant differences in the fatigue intervention effects of olfactory stimuli at different concentration levels, exposure durations, and driving stress conditions?

This study used subjective self-report scales and physiological data to assess drowsiness and fatigue levels in subjects before and after driving simulator testing. Based on the above discussion, the following hypotheses are proposed:

*H1*: Olfactory stimulation has a significant effect on driver fatigue arousal.

*H2*: The stronger the odor, the more significant the driver fatigue arousal.

*H3*: Short-term olfactory stimulation has a significant effect on driver fatigue arousal.

*H4*: Olfactory stimulation under high traffic flow conditions has a significant effect on driver fatigue arousal.

*H5*: The interaction between concentration and duration has a significant effect on driver fatigue arousal.

*H6*: The interaction between concentration and traffic flow has a significant effect on driver fatigue arousal.

*H7*: The interaction between fatigue awakening time and traffic flow has a significant effect on driver fatigue arousal.

## 3 Methods

### 3.1 Experiment design

This experiment was designed as a 2 (fatigue awakening time: 1 min vs. 8 min) × 2 (traffic flow: 800 vehicles/h vs. 1,600 vehicles/h) × 3 (concentration: low, medium, high) mixed experimental design. Independent variables included olfactory stimulus, concentration, and release time. The dependent variable was the degree of arousal, which was represented by KSS, SSS, HRV, and PERCLOS values, respectively.

### 3.2 Participants

From G*Power, it was calculated that the minimum sample size for this experiment should be 30; therefore, 31 participants (M = 35.19, SD = 14.20) were recruited, including 16 males and 15 females. Participants included 15 aged 20–29 years, 7 aged 30–49 years, and 9 aged 50 years or above. All drivers had been issued motor vehicle licenses, and participants had held their driving licenses for an average of 7.26 years (SD = 5.97). Sixteen had been driving for 5 years or less, and 15 had been driving for more than 5 years. All participants had normal vision or corrected vision and were not color blind. All participants were required to sign an informed consent form before participating in the experiment. Before the experiment, participants were asked to sleep no more than 7 h the night before and were not allowed to consume caffeine or alcohol for 12 h prior to the experiment. Therefore, factors that might affect the participants’ normal driving state were excluded to ensure the reliability of the experimental results. This study complied with Chinese laws and regulations regarding scientific research involving healthy human volunteers.

### 3.3 Experimental scenarios and task design

#### 3.3.1 Driving scenario

The experiment was conducted in the afternoon from Monday to Friday to control the potential impact of circadian rhythms on fatigue accumulation. The driving scenario is based on a simulated urban expressway with a total length of approximately 100 kilometers, featuring a two-way, four-lane design with a standard lane width of 3.75 meters. To effectively induce passive task-related fatigue (Schmidt and Bullinger, 2019), the scene is deliberately designed as a highly monotonous environment: the entire road is a straight line, the landscape on both sides consists of repetitive lawns, and the vehicle speed is fixed at 40 km/h (Wang et al., 2017) through a program. Existing studies have shown that such low-task load environments can induce significant fatigue in drivers within approximately 15 min (Qin, 2021; Schmidt and Bullinger, 2019; Jacobé de Naurois et al., 2019).

#### 3.3.2 Odorant

The scent is peppermint, which has been shown to improve lung capacity and inhalation capacity due to the presence of the peppermint aroma, improve oxygen supply to the brain to increase alertness, reduce aggressive driving behavior, and negative emotions such as aggression and stress, while increasing alertness and calmness, and also improve physical activity (Moss et al., 2023). Tisserand pure peppermint essential oil is used to produce the aroma according to Moss et al. (2023). The essential oil is 9 mL and is organic, certified by the Soil Association, and the manufacturing facility has an ISO9001:2015 quality management system with UKAS and European Federation of Cosmetic Ingredients (EFfCI) accreditation, as well as an ISO22716 Good Manufacturing Practice for Cosmetics and an ISO14001 Environmental Management System certification. Batch number 228101 was identified with a Certificate of Analysis by First Natural Brands Ltd., Sussex, UK. The Product Specification Sheet indicates that the essential oil complies with Article 3 (2) (EC) 1,334/2008 Regulation (d) and can therefore be designated as natural and free from any solvents or additives. The parameters are shown in Table 1. The humidifier chosen had a capacity of 100 mL and was placed out of sight of the participants in the test room, with a spray volume of 20 mL/h. The humidifier was turned on when an odor stimulus was required, and a fan was used to blow the air away when the fatigue-inducing phase was entered. According to Chen et al.’s experiment (Chen et al., 2022), the gas mass concentration was 0.66 ~ 0.86 g/m^3^ at low concentration, 3.54 ~ 4.30 g/m^3^ at medium concentration, and 16.50 ~ 21.40 g/m^3^ at high concentration. The ratio of essential oil content in low, medium, and high concentration is about 1:5:25. Therefore, in this experiment, 0.05 mL (1 drop) of essential oil was added to 100 mL of water for making low concentration aromatherapy, 0.25 mL (5 drops) of essential oil was added to 100 mL of water for making medium concentration aromatherapy, and 1.25 mL (25 drops) of essential oil was added to 100 mL of water for making high concentration aromatherapy, with the low, medium, and high concentration of essential oil content set to about 1:5:25.

**Table 1:** Experimental setup and equipment parameters.

Experimental setup	Olfactory stimulation devices		Physiological monitoring devices		Driving simulator
	Humidifier	Peppermint essential oil constituents range (%)	BIOPAC	Head-mounted eye-tracking system	
Equipment parameters	Model: YN24-1	L-menthol: 30–55	Sampling frequency: 50 Hz (system delay <50 ms)	Model: Tobii Pro Glasses 3	Logitech G29 steering wheel
	Water capacity: 100 ml	Trans menthone: 12–33	Collected parameters: real-time electrocardiogram	Calibration: Tobii Glass 3 1.19.1	Logitech G29 accelerator and brake pedal kit
	Fog output: 20 ml/h	1,8-Cineole: 2–10	Host: MP160	Software: Tobii Pro Lab	65-Inch monitor (3,840*2140)
	Power: 4 W	Methyl acetate: 2–10	Acquisition module: ECG100C		Refresh rate: 60 HZ
	Appearance material: ABS	d,l-lsomenthone: 1–6	Lead wire: 1ead110		Brightness: 100%
	Material of water tank: PP	Beta caryophyllene: 0.01–3.5	Electrode patch: EL513		
	Product size: 85*85*150 mm	Limonene: 0.01–3			
	Net weight of the product: 182 g	Neo-menthol: 2–8			
		Menthofuran: 0.01–9			

The concentration ratio (1:5:25) and the corresponding gas mass concentration adopted in this study directly cited the self-verified method (Chen et al., 2022). To ensure the effectiveness of this scheme in this experimental environment, we first strictly reproduced the equipment and environmental parameters of the original method and recruited five volunteers to conduct a pre-experimental sensory test. The results of the single-blind test consistently indicated that the three concentrations could cause significant and clearly graded perceptual differences (low < medium < high), confirming their effectiveness. In the formal experiments, all odor releases were strictly standardized and carried out in a controlled laboratory to ensure the consistency of concentration manipulation. It should be noted that this study did not use a gas concentration meter for real-time monitoring, which is a limitation of this research. However, we believe that through the strict reproduction of the validated protocol and the process control of the system, the odor concentration manipulation in this study is reliable and consistent.

To control the potential influence of expectation effects on subjective evaluations, this study adopted a partially blinded design. Although participants were informed that they might be exposed to odor stimulation during driving, they were completely unaware of the specific concentration groups (low, medium, and high) to which they were assigned. All concentrations of odor stimuli were released using the same equipment and under the same experimental environment, and the experimenter did not provide any suggestive information during each stimulation process. In this way, we strive to minimize the potential expected effects brought about by different concentrations.

#### 3.3.3 Driving simulator

The parameters are shown in Table 1. This study used a static driving simulator, and the experiments were conducted in the laboratory using a medium-fidelity simulator with a 65-inch display (3,840 × 2,160 resolution), a high-performance computer, a steering wheel, brakes, a gas pedal, and a seat, with the seat and steering wheel adjusted to the participant’s measurements. Subjects were tested sitting in a real car seat, using a real steering wheel, with the seat 50 cm from the screen, and the driving simulation scenario *scanner* software was constructed. *Tobii Pro 2* glasses were used to record participants’ eye movements and gaze patterns. The system consisted of glasses with a camera mounted on them, a recording device, and a computer unit to manage the recording process.

### 3.4 Measurement method

To investigate driving fatigue associated with passive tasks, various metrics/indices were used, including objective physiological measures (HRV, PERCLOS) and subjective measures (KSS, SSS). The experiment was divided into eight discrete segments of 15, 1, 15, 8, 15, 1, 15, and 8 min, and HRV and PERCLOS values were calculated within each segment.

#### 3.4.1 Physiological indicators

HRV metrics are obtained by ECG. HRV is measured by changes in heartbeat intervals using a multiconductor ECG connection, reflecting the autonomic regulation of heart rate, which has a strong correlation with fatigue in the psychophysiological field, especially in the driver fatigue field (Mizuno et al., 2011; Sun and Yu, 2014). Many researchers have worked on HRV analysis in both time and frequency domains to assess fatigue (Jiao et al., 2004; Tran et al., 2009; Zhao et al., 2012), and HRV metrics have been prioritized for early fatigue detection (Chowdhury et al., 2018). HRV values are represented by time-domain and frequency-domain metrics. Depending on the different experimental conditions set, the raw data acquired by the MP150 physiological system were processed as follows: (1) Raw ECG data were exported and filtered using AcqKnowledge 5.0 software to calculate HRV. (2) The initial time interval for HRV data processing was set to 10 s, and all the time-domain and frequency-domain metrics were averaged over 10 s for each subject. (3) Select the LF/HF ratio from the processed time- and frequency-domain metrics. (4) The statistical significance of LF/HF was interpreted.

Physiological symptoms were assessed using eye gaze tracking measures. Studies have shown that eye gaze tracking metrics, including percent eyelid closure (PERCLOS) (Wierwille et al., 1994), blink duration (Zargari Marandi et al., 2018), blink frequency (Zargari Marandi et al., 2018), and sweep frequency (Hu and Lodewijks, 2021), are associated with driver fatigue. The following raw eye movement data were recorded: timestamp, eyelid distance, pupil diameter, gaze duration, blink duration, gaze position, and data quality. PERCLOS and blink frequency (BF) were calculated from the raw data to analyze fatigue status. PERCLOS P80 was defined as the proportion of time that the eyes were closed 80% or more during a 1-min interval. Based on empirical evidence, larger PERCLOS and BF indicate higher levels of driver fatigue (Wang et al., 2017; Wierwille et al., 1994; Zargari Marandi et al., 2018), with PERCLOS computed as the maximum opening detected for each participant during each interval of time. The percentage of time the pupil was covered was more than 80%, with higher PERCLOS values indicating higher levels of driving fatigue.

#### 3.4.2 Subjective scale

Driver fatigue was measured by KSS and SSS ratings. The KSS and SSS ratings of all drivers were averaged across eight segments to obtain the average self-rated fatigue level for fatigue-induced and driving arousal across four different conditions. The KSS uses a nine-point ordinal scale ranging from “1. Extremely alert” to “9. Extremely drowsy, unable to stay awake”; the higher the score, the more fatigued the participant felt. The SSS uses a seven-point ordinal scale ranging from “1. Feeling energized, alert, and untired” to “7. Not wanting to try to stay awake, falling asleep quickly, and feeling like dreaming,” with higher scores indicating higher levels of fatigue. The higher the score, the more tired the participant felt.

### 3.5 Experimental procedure

**Figure 1:**
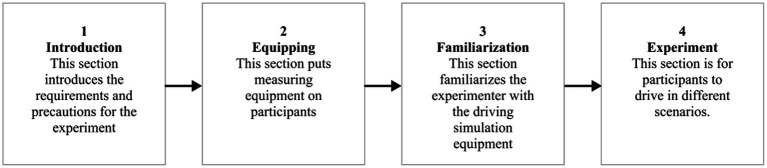
Experimental steps.

**Figure 2:**
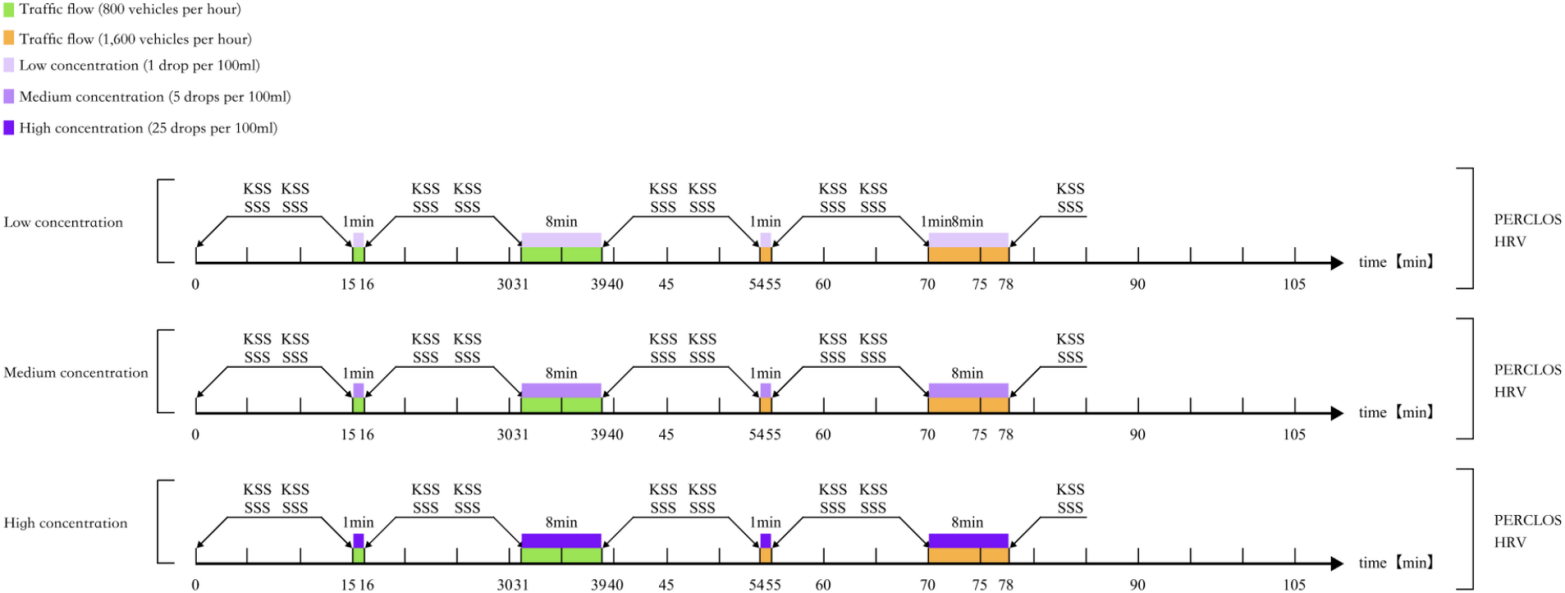
Experimental flow.

The experiment adopted the “fatigue–arousal” paradigm. Each participant was randomly assigned to a fixed mint odor concentration condition (low, medium, or high) and completed four rounds of core tests in sequence. Figures 1, 2 illustrate the steps and flow of the experiment, as follows:

1. Experimental preparation stage. Participants signed the informed consent form and completed the demographic questionnaire, wore physiological signal acquisition devices (ECG and eye tracker), and practiced driving briefly to familiarize themselves with the operation of the simulator. They filled in the KSS (Åkerstedt and Gillberg, 1990) and SSS (Hoddes et al., 1973) scales to obtain the baseline fatigue level.
2. In the formal experimental stage, it is divided into four rounds of core tests. After each 15-min fatigue-induced driving and subsequent olfactory stimulation, participants were required to orally report their subjective fatigue level.

- Conduct the first round of 15-min monotonous driving to induce fatigue. In low-traffic flow, receive a one-minute stimulation with a specified concentration of mint odor.
- Conduct a second round of 15-min monotonous driving to restore the fatigued state. In a low-traffic flow, receive an 8-min stimulation with a specified concentration of mint scent.
- Conduct the third round of 15-min monotonous driving. In high-traffic flow, receive olfactory stimulation of the same concentration for 1 min.
- Conduct the fourth round of 15-min monotonous driving. Receive 8-min olfactory stimulation in high-traffic flow.
- The experiment is over. After all driving tasks were completed, the participants parked safely and removed the physiological signal acquisition devices.

### 3.6 Data processing and statistics

A common method of data analysis for subjective evaluations is to calculate the mean, which is the average of the corresponding scores on the KSS and SSS questionnaires for each stage when added together. We collected 31 subjective data points from each person experiencing 4 fatigue awakening sessions and analyzed the significant effects of pre- and post-awakenings by performing a paired-samples *t*-test on the fatigue awakening scores using SPSS 21.0 software.

Traffic flow (less vs. more) and arousal time (1 vs. 8) were set as within-group terms, and concentration (low vs. medium vs. high) was set as a between-group term. A repeated measures ANOVA was conducted for different dependent variables. The usual analysis of repeated measures ANOVA consists of three steps, namely, between-group effects analysis, sphericity test, and within-group effects analysis. However, if the number of within-group item levels is >2, the test of sphericity is not required, and this test is not needed for this analysis.

**Figure 3:**
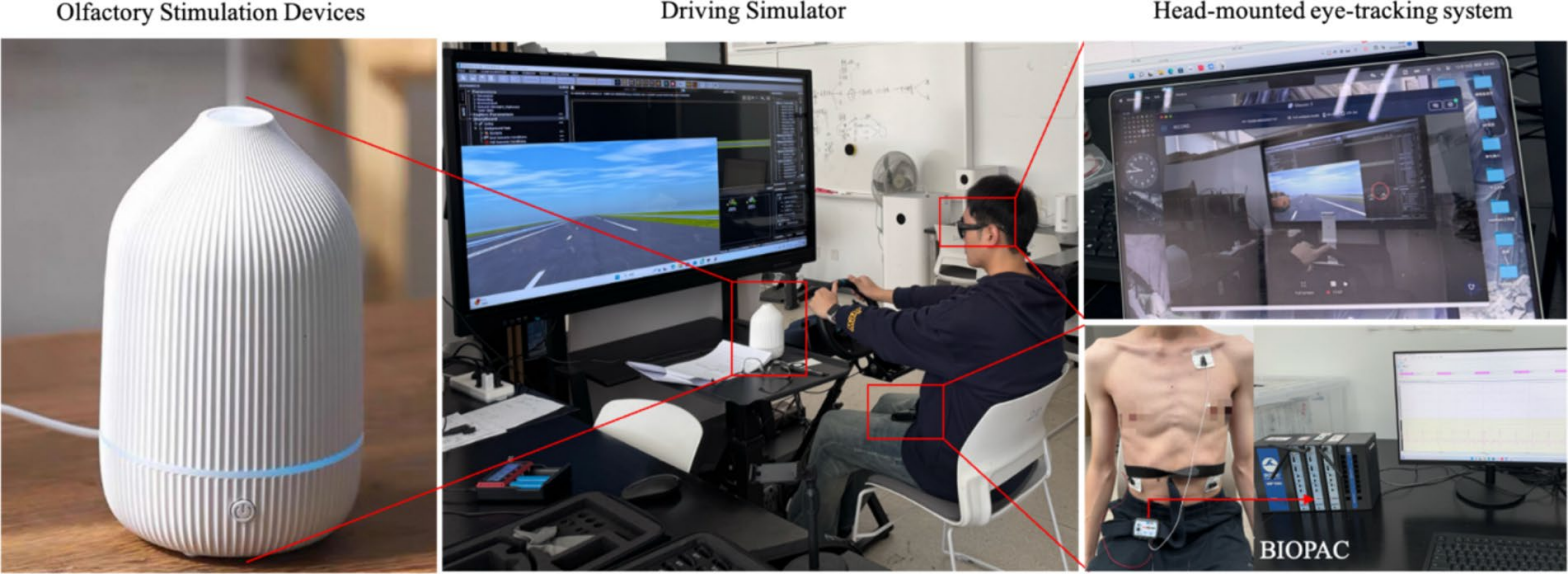
Experimental scene.

Traffic flow (less vs. more) and arousal time (1 vs. 8) were set as within-group terms, and concentration (low vs. medium vs. high) was set as a between-group term. Covariate analysis of covariance was performed by adding covariates (gender, age, and driving age) (Figure 3).

## 4 Results

### 4.1 Pre- and post-effects of odor on fatigue awakening

To analyze the effect of peppermint odor on driver fatigue, the values of the dependent variable should be compared between drivers after fatigue induction and after peppermint arousal to form a paired sample. Therefore, a paired-sample *t*-test was performed for each variable to compare the significance of the differences.

As shown in Table 2, a total of four sets of paired data, all of which show variability (*p* < 0.05). Specific analysis shows that fatigue-evoked KSS and arousal KSS show significance at the 0.01 level (*t* = 15.817, *p* = 0.000), and a specific comparison of the differences shows that the mean value of fatigue-evoked KSS (5.60) is significantly higher than the mean value of arousal KSS (3.00). The fatigue-induced SSS and arousal SSS showed significance at the 0.01 level (*t* = 16.592, *p* = 0.000), and a specific comparison difference shows that the mean value of the fatigue-induced SSS (3.90) is significantly higher than the mean value of the arousal SSS (2.02). The significance between fatigue-evoked LF/HF and arousal LF/HF was presented at the 0.01 level (*t* = 6.106, *p* = 0.000), and a specific contrast difference shows that the mean value of fatigue-evoked LF/HF (2.77) is significantly higher than the mean value of arousal LF/HF (1.23). The significance between fatigue-induced blink frequency and arousal blink frequency was presented at the 0.05 level (*t* = −2.381, *p* = 0.022), and the specific comparative differences showed that the mean value of fatigue-induced blink frequency (34.22) is significantly lower than the mean value of arousal blink frequency (39.36).

**Table 2:** Results of paired *t*-test analysis of four dependent variables before and after arousal.

Name	Paired (mean± standard deviation)		Difference (pair 1 – pair 2)	*t*	*p*
	Pair 1	Pair 2			
KSS pre-post	5.60 ± 1.64	3.00 ± 1.41	2.60	15.817	0.000**
SSS pre-post	3.90 ± 1.32	2.02 ± 0.87	1.87	16.592	0.000**
LF/HF pre-post	2.77 ± 2.93	1.23 ± 1.04	1.54	6.106	0.000**
PERCLOS pre-post	34.22 ± 21.96	39.36 ± 26.73	−5.13	−2.381	0.022*

*p < 0.05, **p < 0.01.

### 4.2 Pre- and post-effects of concentration, duration, and traffic flow on fatigue arousal

Table 3 sets traffic flow (less vs. more) and arousal duration (1 vs. 8) as intra-group terms and concentration (low, medium, high) as inter-group terms, and conducts repeated measures analysis of variance for different dependent variables. Repeated measures analysis of variance (ANOVA) typically consists of three steps: intergroup effect analysis, sphericity test, and intra-group effect analysis. However, if the number of item levels within the group is greater than 2, a sphericity test is not required, and this analysis does not need this test. Due to the presence of demographic variable factors in the variables, covariance analysis (ANCOVA) was conducted again, as shown in Tables 4–6, respectively.

**Table 3:** Means, standard deviations, and sample sizes of subjective evaluation and physiological indicators.

Y	Traffic flow: 800 vehicles/h						Traffic flow: 1600 vehicles/h					
	Fatigue awakening time 1 min			Fatigue awakening time 8 min			Fatigue awakening time 1 min			Fatigue awakening time 8 min		
	Concentration L	Concentration L	Concentration M	Concentration L	Concentration M	Concentration H	Concentration L	Concentration M	Concentration H	Concentration L	Concentration M	Concentration H
KSS	2.900 ± 0.54 (*N* = 10)	3.600 ± 1.35 (*N* = 10)	3.364 ± 1.21 (*N* = 11)	3.800 ± 1.40 (*N* = 10)	3.900 ± 2.28 (*N* = 10)	2.909 ± 1.14 (*N* = 11)	2.700 ± 1.25 (*N* = 10)	3.700 ± 1.49 (*N* = 10)	2.182 ± 0.75 (*N* = 11)	2.600 ± 1.35 (*N* = 10)	2.400 ± 1.65 (*N* = 10)	2.091 ± 0.83 (*N* = 11)
SSS	1.900 ± 0.70 (*N* = 10)	2.500 ± 1.08 (*N* = 10)	2.091 ± 0.54 (*N* = 11)	2.400 ± 0.70 (*N* = 10)	2.600 ± 1.26 (*N* = 10)	2.272 ± 0.65 (*N* = 11)	1.600 ± 0.70 (*N* = 10)	2.300 ± 1.60 (*N* = 10)	1.818 ± 0.40 (*N* = 11)	1.600 ± 0.97 (*N* = 10)	1.700 ± 0.82 (*N* = 10)	1.545 ± 0.69 (*N* = 11)
LF/HF	1.360 ± 0.83 (*N* = 10)	0.731 ± 0.39 (*N* = 10)	1.302 ± 1.00 (*N* = 10)	1.586 ± 0.67 (*N* = 10)	1.293 ± 0.74 (*N* = 10)	2.252 ± 2.47 (*N* = 11)	1.290 ± 0.41 (*N* = 10)	0.760 ± 0.31 (*N* = 10)	1.004 ± 0.87 (*N* = 11)	1.024 ± 0.33 (*N* = 10)	0.874 ± 0.27 (*N* = 10)	1.280 ± 1.06 (*N* = 11)
PERCLOS	39.702 ± 27.46 (*N* = 5)	16.652 ± 14.82 (*N* = 6)	93.18 ± 0 (*N* = 1)	64.010 ± 30.65 (*N* = 5)	30.887 ± 25.65 (*N* = 6)	22.75 ± 0 (*N* = 1)	36.584 ± 24.63 (*N* = 5)	31.825 ± 13.64 (*N* = 4)	45.16 ± 0 (*N* = 1)	47.972 ± 29.43 (*N* = 5)	44.370 ± 17.75 (*N* = 4)	/

**Table 4:** Repeated measures ANOVA of subjective ratings (KSS, SSS) on different independent variables and analysis of covariance.

Y	Variables	*df*	*F*				*p*				Partial eta-squared (partial *η*^2^)			
			RM-ANOVA		ANCOVA		RM-ANOVA		ANCOVA		RM-ANOVA		ANCOVA	
			KSS	KSS	KSS	SSS	KSS	SSS	KSS	SSS	KSS	KSS	KSS	SSS
Subjective questionnaire	Concentration	1	1.224	1.154	4.244	50.211	0.309	0.330	0.017*	0.000**	0.080	0.076	0.072	0.106
	Traffic flow	1	23.897	19.027	12.570	12.921	0.000**	0.000**	0.001**	0.919	0.460	0.405	0.103	0.000
	Fatigue awakening time	2	0.740	0.023	0.303	0.010	0.397	0.879	0.583	0.059	0.026	0.001	0.003	0.050
	Concentration × Traffic flow	1	0.385	0.019	0.203	2.898	0.684	0.981	0.817	0.066	0.027	0.001	0.004	0.031
	Concentration × Fatigue awakening time	2	3.408	2.089	1.397	3.454	0.047*	0.143	0.252	0.987	0.196	0.130	0.025	0.000
	Traffic flow × Fatigue awakening time	2	6.785	7.059	2.729	0.013	0.015*	0.013*	0.101	0.398	0.195	0.201	0.024	0.017
	Concentration × VMT × Fatigue awakening time	2	4.231	0.131	1.701	0.928	0.025*	0.877	0.187	0.938	0.232	0.009	0.030	0.001
	Sex	1			1.709	0.064			0.194	0.838			0.015	0.000
	Age	1			14.507	0.042			0.000**	0.076			0.117	0.029
	Driving age	1			8.861	3.218			0.004**	0.236			0.075	0.013

ANCOVA KSS: *R*^2^ = 0.302, SSS: *R*^2^ = 0.201. *p < 0.05 **p < 0.01.

**Table 5:** Repeated measures ANOVA and ANCOVA of LF/HF for different independent variables.

Y	Variables	*df*	*F*		*p*		Partial eta-squared (partial *η*^2^)	
			RM-ANOVA	ANCOVA	RM-ANOVA	ANCOVA	RM-ANOVA	ANCOVA
LF/HF	Concentration	2	1.709	4.021	0.200	0.021*	0.761	0.069
	Traffic flow	1	10.220	4.634	0.004**	0.004**	0.112	0.041
	Fatigue awakening time	1	2.964	3.424	0.097	0.067	0.275	0.031
	Concentration × VMT	2	1.040	0.508	0.367	0.603	0.099	0.009
	Concentration × Fatigue awakening time	2	1.068	1.235	0.358	0.295	0.072	0.022
	Traffic flow × Fatigue awakening time	1	5.718	2.594	0.024*	0.110	0.073	0.023
	Concentration × VMT × Fatigue awakening time	2	0.131	0.066	0.878	0.936	0.175	0.001
	Sex	1		0.757		0.386		0.007
	Age	1		5.270		0.024*		0.047
	Driving age	1		10.946		0.001**		0.092

ANCOVA *R*^2^ = 0.240. *p < 0.05, **p < 0.01.

**Table 6:** ANCOVA and ANCOVA of repeated measures of different independent variables by PERCLOS.

Y	Variables	*df*		*F*		*p*		Partial eta-squared (partial *η*^2^)	
		RM-ANOVA	ANCOVA	RM-ANOVA	ANCOVA	RM-ANOVA	ANCOVA	RM-ANOVA	ANCOVA
PERCLOS	Concentration	1	2	0.790	2.681	0.404	0.085	0.101	0.156
	Traffic flow	1	1	0.008	2.013	0.930	0.167	0.001	0.065
	Fatigue awakening time	1	1	10.242	1.304	0.015*	0.263	0.594	0.043
	Concentration × VMT	1	2	2.440	2.635	0.162	0.089	0.258	0.154
	Concentration × Fatigue awakening time	1	2	0.116	6.348	0.743	0.005**	0.016	0.305
	Traffic flow × Fatigue awakening time	1	1	1.507	0.030	0.259	0.865	0.177	0.001
	Concentration × VMT × Fatigue awakening time	1	2	0.459	0.341	0.520	0.714	0.062	0.023
	Sex		1		1.785		0.192		0.058
	Age		1		6.627		0.015*		0.186
	Driving age		1		9.428		0.005**		0.245

ANCOVA *R*^2^ = 0.536. 4, 5, 7, 8, 9, 12, 13, 14, 15, 16, 17, 21–31 Missing values excluded. *p < 0.05, **p < 0.01.

#### 4.2.1 Concentration

Table 4 shows that the effect of different concentrations (low, medium, and high) on the dependent variable (KSS) was significant (*p* = 0.017). This means that high concentration leads to smaller KSS compared to low and medium concentrations, so high concentration has a more significant arousal effect than low concentration, while medium concentration, on the contrary, has the worst effect. Table 5 shows that the effect of different concentrations (low, medium, and high) on the dependent variable (LF/HF) is significant (*p* = 0.021). This indicates that medium concentration leads to smaller LF/HF relative to low and high concentrations; thus, medium concentration has a more significant effect on arousal compared to low concentration and the weakest effect compared to high concentration.

#### 4.2.2 Fatigue awakening time

Table 6 shows that the effect of different fatigue awakening times (1 min vs. 8 min) on the dependent variable (wake blink frequency) was significant (*p* = 0.015), indicating that awakening time 1 (1 min) resulted in a smaller wake blink frequency relative to awakening time 2 (8 min). Thus, 1 min of awakening was more significant than 8 min of awakening.

#### 4.2.3 Traffic flow

Table 4 shows that the effect of different traffic flows (800 vehicles/h vs. 1,600 vehicles/h) on the dependent variable (KSS) was significant (*p* = 0.000, *p* = 0.001). This means that traffic flow 1 (800 vehicles/h) leads to a greater KSS relative to traffic flow 2 (1,600 vehicles/h), so more traffic flow has a more significant awakening effect than less traffic flow.

Table 4 shows that the effect of different traffic flows (800 vehicles/h vs. 1,600 vehicles/h) on the dependent variable (SSS) is significant (*p* = 0.000). This indicates that traffic flow 1 (800 vehicles/h) resulted in a greater SSS relative to traffic flow 2 (1,600 vehicles/h); thus, more traffic flow has a more significant awakening effect than less traffic flow.

Table 5 shows that the effect of different traffic flows (800 vehicles/h vs. 1,600 vehicles/h) on the dependent variable (LH/HF) is significant (*p* = 0.004). ANCOVA of Table 5 shows that the effect of different traffic flows (800 vehicles/h vs. 1,600 vehicles/h) on the dependent variable (LF/HF) is significant (*p* = 0.034), indicating that traffic flow 1 (800 vehicles/h) resulted in greater LH/HF relative to traffic flow 2 (1,600 vehicles/h); thus, more traffic flow had a more significant awakening effect than less traffic flow (Table 7).

**Table 7:** Effect of concentration × fatigue awakening time on KSS.

Y	Concentration	Awakening time	Mean difference	Standard error *SE*	*t-*value	*p-*value
KSS	Low	1–8 min	−0.400	0.254	−1.574	0.127
	Medium	1–8 min	0.500	0.254	1.968	0.059
	High	1–8 min	0.273	0.242	1.126	0.270

#### 4.2.4 Interaction effect of concentration × fatigue awakening time

Table 4 shows that the effect of different awakening times (1 min vs. 8 min) on the dependent variable (wake KSS) was not significant at low concentrations. A negative mean difference indicates that awakening time 2 (8 min) resulted in a smaller wake KSS relative to awakening time 1 (1 min). The effect of different awakening times (1 min vs. 8 min) on the dependent variable (wake KSS) was significant (*p* = 0.059) in the case of medium concentration. A positive mean difference indicates that awakening time 2 (8 min) resulted in a greater wake KSS relative to awakening time 1 (1 min); thus, 1 min wake was more effective than 8 min wake at medium concentrations. At high concentrations, the effect of different awakening times (1 min vs. 8 min) on the dependent variable (wake KSS) was not significant. A negative mean difference indicates that awakening time 2 (8 min) resulted in a smaller wake KSS relative to awakening time 1 (1 min).

Table 6 shows that the effect of different concentrations (low, medium, and high) on the dependent variable (arousal blink frequency) was significant (*p* = 0.005) at different fatigue awakening times (1 min vs. 8 min). At awakening time 1, medium concentration resulted in the least blink frequency and the highest arousal; at awakening time 8 min, high concentration resulted in the least blink frequency and the highest awakening.

#### 4.2.5 Interaction effect of concentration × traffic

Tables 4–6 showed that the interaction of concentration and traffic flow was not significant for KSS (*p* = 0.684), SSS (*p* = 0.981), LF/HF (*p* = 0.367), and PERCLOS (*p* = 0.162). The interaction of concentration and traffic flow in ANCOVA was also not significant for KSS (*p* = 0.101), SSS (*p* = 0.066), LF/HF (*p* = 0.110), and PERCLOS (*p* = 0.865).

#### 4.2.6 Interaction effect of traffic flow × fatigue awakening time

Tables 4, 8 show that the effect of different awakening times (1 min vs. 8 min) on the dependent variable (wake KSS) is not significant in the case of low traffic flow (traffic level 1: 800 vehicles per hour). A negative mean difference indicates that awakening time 2 (8 min) resulted in a smaller wake KSS relative to awakening time 1 (1 min). The effect of different awakening times (1 min vs. 8 min) on the dependent variable (awakening KSS) was significant (*p* = 0.018) in the case of high traffic flow (traffic level 2: 1600 vehicles per hour). A positive mean difference indicates that awakening time 2 (8 min) resulted in a greater wake KSS relative to awakening time 1 (1 min); thus, a 1-min awakening is more effective than an 8-min awakening in the presence of heavy traffic.

**Table 8:** Effect of traffic flow × awakening time on KSS, SSS, LF/HF.

Y	Traffic flow	Fatigue awakening time	Mean difference	*SE*	*t*-value	*p*-value
KSS	800 vehicles/h	1–8 min	−0.248	0.203	−1.222	0.227
	1,600 vehicles/h	1–8 min	0.497	0.203	2.444	0.018*
SSS	800 vehicles/h	1–8 min	−0.261	0.143	−1.818	0.074
	1,600 vehicles/h	1–8 min	0.291	0.143	2.029	0.047*
LH/HF	800 vehicles/h	1–8 min	−0.601	0.221	−2.719	0.009**
	1,600 vehicles/h	1–8 min	−0.047	0.221	−0.214	0.831

*p < 0.05, **p < 0.01.

Tables 4, 8 show that the effect of different awakening times (1 min vs. 8 min) on the dependent variable (wake SSS) is not significant in the case of low traffic flow (traffic level 1: 800 vehicles per hour). A negative mean difference indicates that awakening time 2 (8 min) resulted in a smaller wake SSS relative to awakening time 1 (1 min). The effect of different awakening times (1 min vs. 8 min) on the dependent variable (wake SSS) was significant (*p* = 0.047) in the case of high traffic flow (traffic level 2: 1600 vehicles per hour). A positive mean difference indicates that awakening time 2 (8 min) resulted in greater wake SSS relative to awakening time 1 (1 min); thus, a 1-min awakening is more effective than an 8-min awakening in the presence of heavy traffic.

Tables 5, 8 show that the effect of different awakening times (1 min vs. 8 min) on the dependent variable (LH/HF) is significant (*p* = 0.009) in the case of low traffic flow (800 vehicles per hour). A negative mean difference indicates that awakening time 2 (8 min) resulted in a smaller awakening LH/HF relative to awakening time 1 (1 min), and thus an 8-min awakening was more effective than a 1-min awakening in low traffic conditions. The effect of different awakening times (1 min vs. 8 min) on the dependent variable (LH/HF) was not significant in the case of high traffic (Traffic level 2: 1600 cars/h). A positive mean difference indicates that awakening time 2 (8 min) resulted in greater waking LH/HF relative to awakening time 1 (1 min).

#### 4.2.7 Effect of demographic variables on arousal level

Table 9 shows the description of KSS, SSS, LF/HF, and PERCLOS by age and driving age.

**Table 9:** Description of KSS, SSS, LF/HF, and PERCLOS by age.

Y	Age (mean ± standard deviation)			Driving age (mean ± standard deviation)	
	20–29 Years old (*n* = 60)	30–49 Years old (*n* = 28)	50 And over (*n* = 36)	5 Years and under (*n* = 64)	More than 5 years (*n* = 60)
KSS	3.13 ± 1.32	3.43 ± 1.81	2.44 ± 1.03	3.08 ± 1.31	2.92 ± 1.52
LF/HF	1.33 ± 0.79	1.08 ± 0.55	1.21 ± 1.58	1.31 ± 0.77	1.16 ± 1.27
PERCLOS	30.75 ± 25.56	54.41 ± 25.53	32.66 ± 21.45	30.75 ± 25.56	46.85 ± 25.95

In Table 4, the effect of different ages (20–29, 30–49, 50+) on the dependent variable (KSS) is significant (*p* = 0.000). This means that individuals aged 50+ have a smaller KSS relative to those aged 20–29 and 30–49, indicating that the 50 + age group has a more significant arousal effect than the 20–29 age group, while the 30–49 age group has the least effect. In Table 5, the effect of different ages (20–29, 30–49, 50+) on the dependent variable (LF/HF) is significant (*p* = 0.024). This indicates that individuals aged 30–49 have less LF/HF relative to those aged 20–29 and 50+, and that the 30–49 age group has a more significant arousal effect relative to the 50 + age group, while the 20–29 age group has the weakest arousal effect. In Table 6, the effect of different ages (20–29, 30–49, 50+) on the dependent variable (blink frequency) was significant (*p* = 0.015). This indicates that individuals aged 20–29 have less blinking frequency relative to those aged 50+ and 30–49, and that the 20–29 age group has a more significant arousal effect relative to the 50+ age group, while the 30–49 age group has the weakest arousal effect.

In Table 4, the effect of different driving ages (5 years and below, 5 years and above) on the dependent variable (KSS) is significant (*p* = 0.004). This indicates that 5 years and below resulted in greater KSS relative to 5 years and above; thus, the driving age of 5 years and above had a more significant arousal effect than 5 years and below. In Table 5, the effect of different driving ages (5 years and below, 5 years and above) on the dependent variable (LF/HF) is significant (*p* = 0.004). This indicates that 5 years and below resulted in greater LF/HF relative to 5 years and above; thus, the driving age of 5 years and above had a more significant arousal effect than 5 years and below. In Table 6, it indicates that 5 years and below causes greater blinking frequencies relative to 5 years and above; therefore, the driving age of 5 years and below has a more significant arousal effect than 5 years and above.

## 5 Discussion

In this study, the effect of different awakening times, traffic flow, concentration, age, driving age, and gender on the effect of fatigue awakening was systematically assessed using subjective variables (KSS, SSS scores), HRV data, and PERCLOS data. Moderation of all independent variables significantly reduced subjects’ fatigue, with significant changes in KSS, SSS, and HRV data, thus supporting the H1 hypothesis. The arousal effect of concentration was subjectively perceived as high concentration being more significant than low concentration, with medium concentration being the least effective. However, physiological measurements indicated that medium concentration was more effective for arousal than low concentration, and high concentration had the weakest arousal effect, leading to the conclusion that the H2 hypothesis was not valid. Physiological measures of arousal time indicated that 1-min arousal had a more significant effect than 8-min arousal, thus supporting hypothesis H3. Both subjective analysis and physiological measurements showed that more traffic had a more significant arousal effect than less traffic. For a given arousal time, the arousal effect of more traffic was significantly better than that of the less traffic condition, especially in the 8-min arousal; the degree of arousal was higher in the 8-min arousal with more traffic than in the 8-min arousal with less traffic. Therefore, more traffic would lead to faster arousal, supporting hypothesis H4. The interaction between concentration and awakening time on wake effects was more significant for 1-min wake than 8-min wake at moderate concentrations. Only in the 1-min awakening with high traffic did medium concentration perform significantly better than high concentration, while low concentration had the worst effect. At 1-min awakening time, medium concentration resulted in the least blinking frequency and the highest degree of arousal; at 8-min awakening time, high concentration resulted in the least blinking frequency and the highest degree of arousal. This shows that the arousal effect of the high-concentration state is not as effective as it should be and indicates that it is more appropriate to maintain a moderate range of concentration to achieve an optimal state of arousal. Therefore, hypothesis H5 is supported. The interaction of traffic flow and concentration was not significant, indicating that concentration did not significantly modulate the effect of traffic flow; thus, hypothesis H6 is not valid. Regarding the effect of traffic flow and awakening time on the effect of awakening, subjective analysis indicated that 1-min awakening was more effective than 8-min awakening with more traffic; however, HRV data analysis indicated that 1-min awakening with more traffic was the best, as the LF/HF value was significantly reduced after awakening, suggesting that the awakening measure was effective in improving the balance of the autonomic nervous system. Physiological measurements indicated that 8-min awakening was better than 1-min awakening with less traffic. Therefore, hypothesis H7 is supported. The subjective perception of age showed that arousal was more effective at age 50 + than at age 20–29, while it was least effective at age 30–49. Physiological measures of age showed that the arousal effect was more significant at age 30–49 relative to age 50 + and weakest at age 20–29. Therefore, hypothesis H8 is not valid. The arousal effect of gender was not significant overall; thus, gender differences had no effect on arousal from driving fatigue, and hypothesis H9 was not valid. The interaction of age and gender was not significant, indicating that age and gender did not significantly modulate the effect of odor on driving arousal; therefore, hypothesis H10 was not valid. Both subjective perceptions of driving age and electrocardiographic measures showed that a driving age of 5 years or more had a more significant arousal effect than 5 years or less, but blink frequency was the opposite.

### 5.1 Effect of concentration on arousal effect

The arousal effect of concentration was subjectively perceived as more significant for high concentration than low concentration, which is consistent with the findings of Baccarani et al. (2021) that, for all odors, higher concentration produced higher perceived intensity and higher stimulus judgments. However, physiological measurements yielded a more significant arousal effect of medium concentration for low concentration and the weakest arousal effect for high concentration. Inconsistent with Hou et al. (2022) study, the highest and lowest concentrations were relatively easy to recognize, consistent with McClintock et al. (2020) study, where some highly sensitive receptors responded only to low concentrations compared to high concentrations, and with the DeChant and Hall (2021) study, which found that explicit training of canines for lower concentrations is essential for generalized trace odor detection. This separation between subjective and objective reports, to a certain extent, reduces the possibility that research results are entirely driven by the expected effect. If the results are dominated only by the expected effect, the subjective and objective data should show a consistent trend. This may be because high concentration stimuli may elicit a stronger perceptual response in a short period of time, causing subjects to subjectively perceive a more significant arousal effect, but this perception may not be fully consistent with actual physiological recovery. This reveals the complex relationship between perception and physiological response during the process of olfactory arousal. In addition, physiological indicators such as HRV reflect the activity of the autonomic nervous system and can more objectively assess the fatigue arousal effect. Medium-concentration stimulation may be more conducive to restoring autonomic nervous system homeostasis, whereas high-concentration stimulation may lead to overactivation and inhibit physiological recovery instead. When designing arousal interventions, it may be necessary to select the appropriate stimulus intensity based on specific application scenarios (e.g., driving safety and workplace fatigue management). This finding provides an important rationale for the design of fatigue interventions—short-term emergency scenarios may use high stimulus concentrations to achieve rapid arousal, whereas persistent fatigue management requires the selection of moderate concentrations to maintain a normal state. Future studies could incorporate more physiological (e.g., EEG and galvanic skin response) and behavioral (e.g., reaction time and error rate) metrics to comprehensively assess arousal effects.

### 5.2 Effect of fatigue, awakening time, and traffic flow on awakening effect

Physiological measurements of the effect of awakening time on arousal concluded that a 1-min wake was more effective than an 8-min wake. Both subjective analyses and physiological measures showed that more traffic had a more significant arousal effect than less traffic. For a given arousal time, the arousal effect of more traffic was significantly better than that of the condition with less traffic, especially in the 8-min arousal, where the degree of arousal was higher in the 8-min arousal with more traffic than in the 8-min arousal with less traffic; thus, more traffic led to faster arousal.

The results of this study indicate that subjectively, 8-min arousal is considered to be more effective than 1-min arousal regardless of the amount of traffic, and objective data indicate that 1-min arousal is more effective in heavy traffic. This finding is consistent with the study by Yoshida et al., who concluded that odor release for 1 min and fluctuating release between 5 and 9 min was the most effective method. However, it is inconsistent with the findings of Pujiartati et al. (2019), who concluded that both short- and long-term arousal were effective in reducing driver fatigue. The study revealed a context-dependent optimization of the duration of arousal stimuli in the driving environment, where subjective assessments were biased in favor of long-duration stimuli by cognitive bias, whereas objective performance metrics showed that short-duration stimuli exhibited superior effects under high traffic pressure due to better temporal synchronization (Salvucci, 2006) and avoidance of sensory adaptation (Dalton, 2000). This subject–object separation phenomenon is consistent with Wickens (2002) multiresource theory, which states that matching the temporal structure of the stimulus duration to the task demands is more important than the absolute duration in complex task scenarios. Practically, it provides a scientific basis for the design of intelligent in-vehicle awakening system, and it is suggested that short-duration (1-min) odor stimulation should be used in high traffic load sections, while it can be appropriately prolonged up to 8 min in low load sections to achieve the optimal fatigue intervention effect.

### 5.3 Effect of concentration and arousal time interaction effect on arousal effects

The 1-min awakening was more effective than the awakening at medium concentrations. Only in the 1-min awakening with high traffic did medium concentration prove significantly better than high concentration, while low concentration was the least effective. At 1-min awakening, medium concentration resulted in the lowest blink frequency and the highest awakening, while at 8-min awakening, high concentration resulted in the lowest blink frequency and the highest awakening. The results suggest that the optimization parameters for the driving arousal stimuli follow neuroeconomic principles, with medium concentrations achieving the best results by balancing the Yerkes–Dodson arousal curves (Sarter et al., 2001) with cross-modal resource allocation (Spence et al., 2000) in the short-duration intervention (1 min); whereas the long-duration intervention (8 min) requires a higher concentration to overcome olfactory adaptation (Dalton, 2000). This non-linear concentration–duration interaction corroborates Hancock’s theory of situational awareness (Hancock, 2018) that stimulus parameters need to dynamically match the temporal structure of the task load. On the practical level, this finding provides an important basis for parameter optimization of intelligent in-vehicle arousal systems: medium-concentration short-duration (1-min) stimuli should be used for quick refreshment during peak hours, while high-concentration intermittent (8-min) stimuli are required to maintain alertness during long-distance driving; on the theoretical level, the study verifies that the optimal arousal parameter for a driving environment depends on the dynamic balance between task duration and cognitive load.

### 5.4 Impact of demographic variables

The insignificant effect of gender on arousal effects is inconsistent with the findings of Kim and Song (2022), Chmiel and Schubert (2017), Cometto-Muñiz and Abraham (2008), and Koelega and Köster (1974). Reports are consistent. However, Glass and Heuberger’s study found that age had a significant effect on olfactory sensitivity, with older individuals suffering from olfactory decline (Glass and Heuberger, 2016). The arousal effect of the 30–49 age group in the present study was significantly better than that of the 50 + age group, in line with their study. The subjective arousal advantage of the group over 50 years old may be related to higher perceptual novelty and significance. With the decline of olfactory sensitivity caused by aging (Glass and Heuberger, 2016), a clearly perceptible odor stimulus becomes more prominent, and this prominent perception may amplify their subjective sense of wakefulness. The optimal physiological arousal exhibited by the 30–49 age group may reflect the unique advantages of this age group. Based on the Optimal Arousal Theory (OAT), we have extended this and proposed that middle-aged drivers aged 30–49 may be at an optimal balance point between sensory and physiological functions: they maintain relatively complete olfactory perception ability, and their autonomic nervous system’s response to moderate stimuli also maintains the best flexibility, thus achieving the optimal arousal state at the physiological level. Of course, this explanatory framework still needs to be further verified and refined by future research. The arousal effect was higher for 30–49 year-olds than for 50 year olds and older and for 20–29 year-olds, consistent with Glass and Heuberger (2016), younger people (≤40 years) were less positively emotional and slightly less alert compared to older people (≥61 years), which may be related to younger people’s or children’s experiences at school. The non-significant effect of the interaction of gender and age on fatigue arousal can be explained through Herb’s optimal arousal theory (OAT) and Berlyne’s theory by the fact that the level of arousal is related to hedonic experience. There are personality differences that are shown in specific environments or certain olfactory tasks and are not shown in the driving task.

Both subjective perceptions of driving age and electrocardiographic measurements showed that arousal was more pronounced with more than 5 years of driving experience than with 5 years or less, but blinking frequency was the opposite. First, subjects were asked after the experiment whether the reason could be visual fatigue due to staring at the electronic screen for a long time, rather than driving fatigue caused by traveling, which is one of the limitations of the practical and experimental simulations. Although the experiment was conducted in a simulated driving scenario, there are still differences from the real driving environment. Future research could further validate the results in a real driving environment. Second, eye movement control is at a higher level of automation, and blink modulation is controlled more by the basal ganglia than by the prefrontal lobe, leading to a dissociation of behavioral metrics from arousal at the conscious level. Consistent with a study by Calvo and Lang (2004), autonomic responses (e.g., heart rate) dissociated from conscious reports in a high proficiency task, suggesting the phenomenon of neural dissociation in automated behavior. The study reveals a hierarchical character of the modulation of the olfactory arousal effect by driving experience, whereby, at the level of autonomic nervous system activation and subjective perception, the experience advantage is reflected in a stronger stimulus response; whereas, at the level of automated behavioral regulation, adaptive mechanisms developed by prolonged driving may produce a qualitative change in the response pattern, possibly reflecting a paradigm shift in neuromodulation triggered by driving experience. That is, blink modulation by prolonged training is shifted from prefrontal lobe-dominated active control to basal ganglia-mediated automated processing (Graybiel, 2008), leading to a dissociation of behavioral indicators from arousal at the conscious level (Shiffrin and Schneider, 1977).

Age differences may be prioritized over gender differences in the development of pervasive olfactory arousal protocols, and the development of more intense olfactory stimulation protocols for older drivers in particular still requires attention to the presence of individual differences; the theoretical level validates the mechanisms by which age-related olfactory decline affects driving safety. Neuroadaptive revelations demonstrated by experienced drivers require a reassessment of the validity of traditional fatigue monitoring metrics.

### 5.5 Limitations and future research

However, this study still has some limitations. First, the experimental environment is limited. In the blink frequency measurements, it was found that there was an arousal effect for the 1-min arousal with a lot of traffic and the 1-min arousal with a little traffic, while the 8-min arousal with a little traffic and the 8-min arousal with a lot of traffic instead led to a significant increase in blink frequency. This is inconsistent with the results of subjective and HRV measurements, and subjects were asked after the experiment if the reason may be visual fatigue caused by staring at the electronic screen for a long time rather than driving fatigue caused by traveling, which is one of the limitations of the practical and experimental simulations that exist. Although the experiment was conducted in a simulated driving scenario, there are still differences from the real driving environment. Future studies can further validate the results in real driving environments. Second, although the total sample size is sufficient to test the main within-subject effects of traffic flow and stimulus duration, the subsequent division into concentration subgroups led to a smaller sample size for each specific concentration level (low, medium, and high), which may limit the statistical ability to detect smaller interaction effects involving concentration factors. Therefore, it needs to be verified in larger-scale studies in the future. In addition, due to objective factors, data loss occurs in the measurement of blink frequency, reducing the effective sample size of this specific indicator, which may affect the universality of the PERCLOS results. Future research should recruit a larger sample population to ensure the reliability of subgroup analysis and verify the stability of research results, especially high-order interactions. Third, there is a single type of arousal stimulus. Only a single type of arousal stimulus was used in this study, and the effect of combining multiple stimuli (e.g., visual, auditory, and tactile) could be explored in the future. Fourth, long-term effects were not assessed. The present study only evaluated the arousal effect within 10 min, and the longer-term (e.g., 1 h and above) effects of arousal measures and their impact on driving safety can be further explored in the future.

Future studies need to expand the sample size to improve statistical efficacy; consider introducing other variables that may affect the dependent variable, such as education level and driving habits; try other statistical methods or models (e.g., mixed-effects modeling) to improve the fit; further explore the mechanism of the effect of age on 1-min arousal of fewer traffic flows, and explore in depth the mechanism of the traffic flow× concentration interactions to clarify how concentration moderates traffic flow.

## 6 Conclusion

This study aimed to investigate the effects of different awakening times, traffic flows, and concentrations on the effectiveness of fatigue awakening measures, which were systematically assessed by subjective variables (KSS, SSS scores), HRV data, and PERCLOS data. The experimental design included two awakening times (1 min and 8 min), two traffic flows (low and high traffic), and three concentration levels (low, medium, and high). The study comprehensively assessed the arousal effect and its influencing factors using paired-samples *t*-tests, repeated-measures analysis of variance, and analysis of covariance, combined with subjective ratings and physiological indicators.

This study systematically evaluated the effects of awakening time, traffic flow, and concentration on the effects of fatigue awakening, revealing that the 8-min awakening was most effective under conditions of high traffic flow, that concentration had a limited effect on the awakening effect, and that the HRV indicator was more sensitive to the awakening effect, whereas the sensitivity of the PERCLOS indicator was lower.

This study enriched the theoretical framework of fatigue arousal. It systematically assessed the arousal effect through multidimensional indicators (subjective score, HRV, PERCLOS), which provided a new theoretical perspective for fatigue arousal research. The study revealed the interaction between arousal time and concentration, finding that arousal time and concentration had a significant interaction effect on the arousal effect, which provided a theoretical basis for understanding the relationship between the internal environment and arousal measures. It expanded the study of the effect of concentration on arousal effect. Although the effect of concentration on arousal effect is limited, this study revealed the advantage of medium concentration under specific conditions, providing new research directions for the future. This study provides a theoretical basis for understanding the relationship between the internal environment and arousal measures. It also expands the study of the effect of concentration on arousal effect. Although the effect of concentration on arousal effect is limited, this study reveals the advantage of medium concentration under specific conditions, which provides a new direction for future research.

The results of this study show that an 8-min awakening is the most effective, providing an important reference for designing driving fatigue warning systems. For example, an 8-min awakening strategy can be used to optimize driver fatigue recovery in highway low-traffic environments. In highway peak commute and other traffic flow situations, a 1-min awakening decreases the driver’s subjective fatigue, while an 8-min awakening keeps the driver physiologically alert. The combination of the two reduces driving fatigue and accidents. The results of the study can provide a basis for fatigue management in high-intensity work environments (e.g., logistics and healthcare). For example, the use of medium-intensity arousal stimuli during work breaks may be more effective in improving employee fatigue. The effect of age on arousal effectiveness suggests that individualized fatigue intervention programs could be designed for different age groups in the future, such as longer arousal measures for people over 50 years of age.

## Data Availability

The raw data supporting the conclusions of this article will be made available by the authors, without undue reservation.
